# Experimental Study of a Quad-Band Metamaterial-Based Plasmonic Perfect Absorber as a Biosensor

**DOI:** 10.3390/molecules27144576

**Published:** 2022-07-18

**Authors:** Semih Korkmaz, Evren Oktem, Ramin Yazdaanpanah, Serap Aksu, Mustafa Turkmen

**Affiliations:** 1 Department of Computer Engineering, Bandirma Onyedi Eylul University, Balikesir 10200, Turkey; semihkorkmaz@bandirma.edu.tr; 2 Biomedical Science and Engineering, Koc University, Istanbul 34450, Turkey; eoktem21@ku.edu.tr; 3 Materials Science and Engineering, Koc University, Istanbul 34450, Turkey; ryazdaanpanah19@ku.edu.tr; 4 Department of Physics, Koc University, Istanbul 34450, Turkey; 5 Department of Electrical and Electronics Engineering, Erciyes University, Kayseri 38039, Turkey; murkmen@gmail.com; 6 Fotonik Technology and Engineering Co. Inc., Erciyes Technopark Co., Kayseri 38010, Turkey

**Keywords:** perfect absorber, quad-band resonances, refractive index-based sensing, photochemical immobilization technique, S100A9

## Abstract

We present a metamaterial-based perfect absorber (PA) that strongly supports four resonances covering a wide spectral range from 1.8 µm to 10 µm of the electromagnetic spectrum. The designed perfect absorber has metal–dielectric–metal layers where a MgF_2_ spacer is sandwiched between an optically thick gold film and patterned gold nanoantennas. The spectral tuning of PA is achieved by calibrating the geometrical parameters numerically and experimentally. The manufactured quad-band plasmonic PA absorbs light close to the unity. Moreover, the biosensing capacity of the PA is tested using a 14 kDa S100A9 antibody, which is a clinically relevant biomarker for brain metastatic cancer cells. We utilize a UV-based photochemical immobilization technique for patterning of the antibody monolayer on a gold surface. Our results reveal that the presented PA is eligible for ultrasensitive detection of such small biomarkers in a point-of-care device to potentially personalize radiotherapy for patients with brain metastases.

## 1. Introduction

Perfect absorbers (PAs) provide near unity absorption by critical coupling when dissipative loss is equal to the rate of radiative loss [1,2,3]. Enhanced coupling can be attained by matching the incident radiation to the radiation pattern of a localized surface plasmon resonance (LSPR), thus light scattering in all directions can be eliminated [4]. PAs are designed as three layers that consist of a dielectric interlayer sandwiched between two metal structures [5,6]. As the bottom metal film reflects all signals, the top metal structure absorbs most of the incident radiation at the specific resonance frequencies according to the shape and length of resonator [7,8,9,10]. In these structures, dielectric spacer layer thickness is crucial to obtain impedance matching to reach maximum absorption level [11]. Locating nanoantennas on multilayered structures provides a narrower full width at half maximum (FWHM) as compared to a monolayer one [11]. Narrowband features of PAs with high absorption and near-field enhancement make them ideal devices for many light-matter interaction applications such as biosensing, infrared spectroscopy and photodetection [12,13,14,15,16]. When a metal-based resonator is illuminated by a light source, free electrons inside the metal start to oscillate, resulting in surface plasmons [17]. The surface plasmons move along the metal surface by coupling with the incident electromagnetic wave [17,18,19]. Due to fact that the lifetime of surface plasmons is so limited, metamaterial-based nanoantennas are designed periodically to increase that time [20,21,22]. Manipulating and absorbing light at a desired wavelength can be utilized to improve applications from the ultraviolet to infrared regime of the electromagnetic spectrum [23,24,25,26,27]. The use of multiscale structures provides the excitation of multiple resonances. Top-down nanofabrication techniques have allowed researchers to design multispectral particle-, aperture- and PA-based nanoantennas [28,29,30,31,32]. However, designing and controlling multi-resonant nanoantennas has some challenges, as resonances can couple with neighbor resonances and result in lower intensity. The usability of resonances can be limited [33].

In this work, we manufacture and optically study a quad-band PA composed of gold nanoantennas operating in IR frequencies and test its sensing capacity for a clinically relevant S100A9 biomarker for brain metastatic cancer cells. Numerical simulations are performed via the finite-difference time-domain (FDTD) method using commercial software (Lumerical FDTD Solutions) [34]. We experimentally obtain multi-band resonances with high absorption values between 80% and 90%. At resonance frequencies, the PA platform shows near-field intensity (|E^2^|/|E_incident_^2^|) that is enhanced 6500-fold over a large number of hot-spots that are available on the PA, as the biomolecules are free to stick on those hot spots. We numerically and experimentally investigate the refractive index sensing ability of the manufactured PA. All the resonances contribute to the sensing and the PA presents a theoretical refractive index sensitivity (S = Δλ/Δn) as large as 3000 nm/RIU. The experimental molecular sensing of the 14 kDa/nm-thick S100A9 antibody is also demonstrated.

## 2. Numerical and Experimental Design of Quad-Band PA

**Figure 1:**
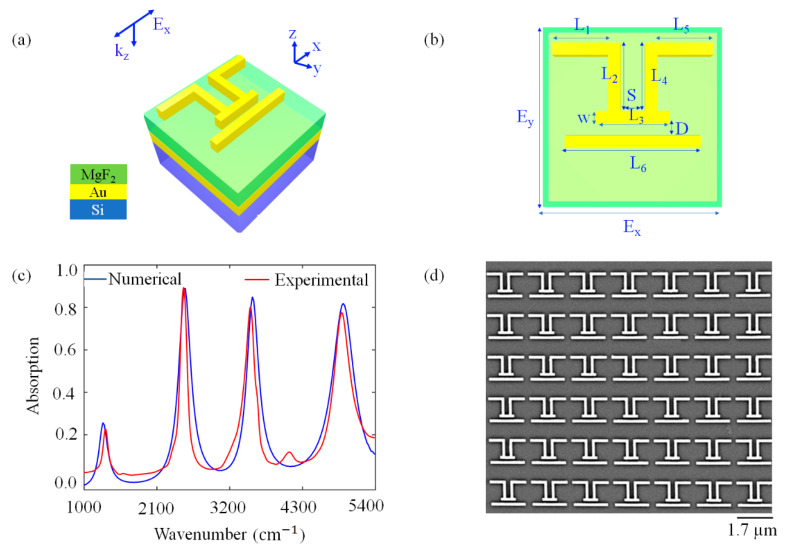
Schematic view of designed structure; (**a**) 3D view of the unit cell and (**b**) top view. (**c**) Simulated and experimental absorption spectrum of the PA under x-polarized illumination source. The results are nearly identical in terms of bandwidth and resonance frequency. (**d**) SEM image of the fabricated nanoantenna arrays that gives the optical responses presented in (**c**).

We studied a novel geometry that obtains the highest number of accessible surface-enhanced areas and allows the maximum light absorption. Figure 1a illustrates the schematic view of the multilayered structure that is defined as a perfect absorber. The design was formed using a compact settlement of multiple rod-shaped antennas oriented in the x- and y-direction to obtain the most efficient antenna coupling that enables quadruple resonances within only the infrared frequency range. This platform consisted of a 100 nm-thick MgF_2_ spacer between patterned 90 nm-thick gold (Au) nanoparticles and a 200 nm-thick Au film. These layers were located on the silicon (Si) substrate with the thickness of 0.5 µm. The thickness of the MgF_2_ spacer is critical to obtain near unity absorption and was studied elsewhere [8,11,13]. Infrared light was applied along the x-direction (Figure 1a) for all analyses since the designed structure is polarization sensitive. Discrete quad-band resonant behavior was obtained only when the polarization of the illumination source was aligned with the *x*-axis direction as compared with other transversal polarizations. The frequency-dependent dielectric constants of the metals were taken from Palik [35] and dielectric constants of MgF_2_ were taken from Dodge [36]. Periodic boundary conditions were along the *x*- and *y*-axes and the perfectly matched layers were along the *z*-axis. The top view of the PA is presented in Figure 1b. We determined the lengths of the rods as L_1_ = L_5_ = 550 nm, L_2_ = L_4_ = 700 nm, L_3_ = 600 nm, L_6_ = 1200 nm, S = 200 nm, D = 100 nm and w = 100 nm, and the array periodicity was 1.7 µm along the x- and y-axes. After deciding the optimum geometric parameters for perfect absorption, we patterned the nanoparticles using electron-beam lithography (EBL) and the standard lift-off process. The manufacturing steps were as follows: first, the 200 nm-thick Au film and 100 nm-thick MgF_2_ dielectric spacer were deposited onto the Si substrate by using electron-beam evaporation. We chose MgF_2_ due to its high optical quality in an infrared regime and its easy deposition conditions in an e-beam evaporation device under similar pressure values as gold. Then, polymethyl methacrylate (PMMA), as a positive photoresist, was coated on top of the MgF_2_ layer by spin-coating at 2500 rpm to obtain ideal 200 nm thickness. EBL was applied on this surface to transfer patterns of the nanoantennas. By passing a chip through a methyl isobutyl ketone–isopropyl alcohol (MIBK–IPA (1:3)) solution for 50 s, we removed the unexposed area. Then, 3 nm-thick titanium (Ti) and 90 nm-thick Au were deposited by e-beam evaporation. Finally, as a standard lift-off process, we applied acetone solution at 80 °C for 35 m to obtain nanoantennas clearly. The nanoantennas were patterned over an area of 85 μm × 85 μm. The resulting PA devices were characterized optically by the Bruker Hyperion 2000 Fourier-transform infrared (FTIR) setup to obtain absorption spectra of the nanoantennas. The spectra were collected with 4 cm^−1^ resolution, consisting of 1024 scans co-added with a mirror repetition rate of 20 kHz. Figure 1c shows numerical and experimental absorption spectra of the PA. The numerical and experimental absorption spectra results are nearly identical in terms of absorption and resonance frequencies. This PA platform supported four absorption peaks at 1283, 2529, 3542 and 4936 cm^−1^. In both simulations and experiments, we obtained nearly 30% absorption for the first resonance and changing between 80% and 90% absorption for the other resonances. All resonant modes were well-defined. We observed a mode with low intensity at around 4000 cm^−1^ due to the presence of an adhesion layer of Ti. The small deviations from the simulation and the experimental results can be attributed to the geometric differences, such as the curved corners of the manufactured nanostructures compared to the sharp edges designed in the simulations [11,14,26] and optical parameters that are used for single-crystalline materials in simulations. We should also note that in this work, we used a commercially available FDTD solver, which is a discrete mesh-based electromagnetic solver, instead of a continuous one. Figure 1d shows the scanning electron microscope (SEM) image of the fabricated nanoantenna array. The uniform patterning of nanoantennas is observed over the full patterned area.

## 3. Results and Discussion

### 3.1. Near-Field Intensity Analyses of the Quad-Band PA

**Figure 2:**
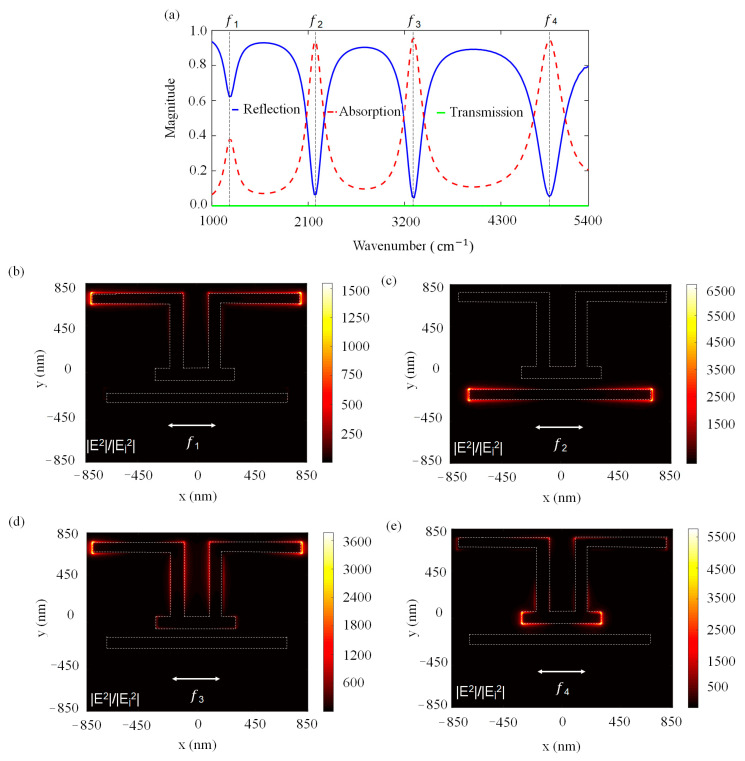
(**a**) Simulated reflection, absorption and transmission spectra of the PA and near-field intensity distributions on Au nanoantennas for: (**b**) the first mode at 1283 cm^−1^, (**c**) the second mode at 2529 cm^−1^, (**d**) the third mode at 3542 cm^−1^, (**e**) the fourth mode at 4936 cm^−1^. Array periodicity is 1.7 µm. The scale on the right shows the maximum near-field intensity calculated on the antenna surface for the corresponding frequency of each subplot.

It is crucial to investigate the near-electric field distribution of the presented PA as the enhancements are the primary indicator for its molecular sensing capacity. Figure 2a indicates the calculated absorption (A), reflection (R) and transmission (T) spectra of PA for L_1_ = L_5_ = 600 nm, L_2_ = L_4_ = 700 nm, L_3_ = 600 nm, L_6_ = 1400 nm, S = 200 nm, D = 100 nm, and w = 100 nm. The absorption spectrum is obtained via A = 1 – R − T. The transmission is zero as the thick Au film behaves like an optical mirror and reflects all signals, thus the absorption is equal to 1 − R. The PA exhibits a quad-band spectral behavior with four plasmonic modes at f_1_ = 38.46 THz, f_2_ = 75.81 THz, f_3_ = 106.18 THz, and f_4_ = 147.98 THz. Figure 2b–e illustrate the near-electric field intensity enhancement distributions (|E|^2^/|E_incident_|^2^) for each resonance frequency under the x-polarized light source. In Figure 2b, it is observed that the first resonant mode at f_1_ originates from L_1_ and L_5_ nanorods located along the *x*-axis with an enhancement factor as large as 1500. In Figure 2c, it is clearly seen that L_6_ nanorods generated the second resonant mode. The third resonant mode is revealed by L_1_, L_2_, L_4_ and L_5_ in Figure 2d. The fourth resonant mode at f_4_ provides local electromagnetic fields mainly concentrated at the arms of L_3_ along the *x*-axis in Figure 2e. There is a good relationship between the absorption and surface enhancement factors. When the absorption is nearly 40%, the near-field intensity reaches a 1500-fold enhancement factor for the first mode. When the absorption is 90%, a 6500-fold enhancement is obtained for the second mode.

### 3.2. Numerical and Experimental Spectral Tunability of Quad-Band Resonances

**Figure 3:**
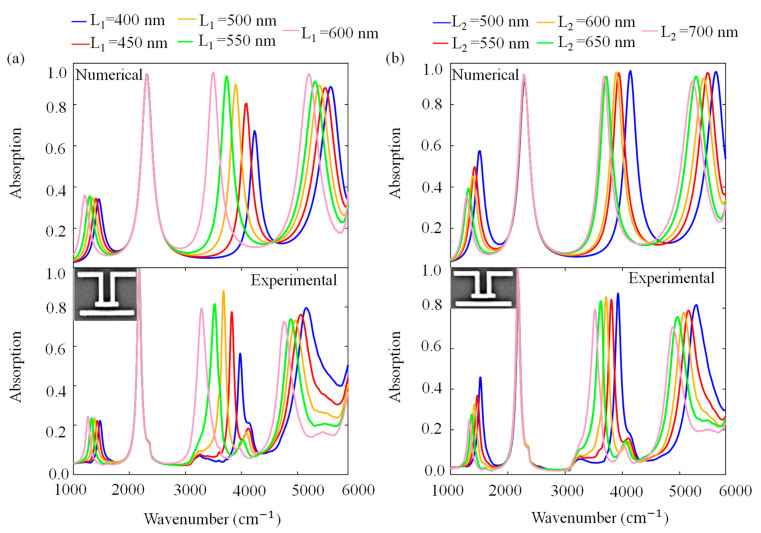
Absorption spectrum of the PA platform for (**a**) numerical and experimental L_1_ sweep; (**b**) numerical and experimental L_2_ sweep under the x-polarized light source. The insets show the SEM image of unit cell for L_1_ = 400 nm and L_2_ = 550 nm. Array periodicity is 1.7 µm.

The high tunability performance of the quad-plasmonic resonant modes is shown by a length sweep. We performed a systematic length-tuning over the designed PAs and investigated the dependence of the absorption spectrum on lengths of rods numerically and experimentally. Figure 3 shows the numerical and experimental parameter sweep for L_1_ and L_2_ with a step size of 50 nm. Figure 3a illustrates the variations in the numerical and experimental absorption spectrums for different L_1_ values while keeping the other parameters constant at L_2_ = L_4_ = 700 nm, L_3_ = 600 nm, L_6_ = 1200 nm, S = 200 nm, D = 100 nm and w = 100 nm. As L_1_ increases, the first, third and fourth resonance modes shift to lower frequencies. For the third mode, increasing L_1_ provides higher absorption values due to the fact that near-field coupling increases between periodic rods. Figure 3b presents numerical and experimental absorption spectra for different *L_2_* values when the other lengths are L_1_ = L_5_ = 550 nm, L_3_ = 600 nm, L_6_ = 1200 nm, S = 200 nm, D = 100 nm and w = 100 nm. When L_2_ increases, the first, third and fourth resonance modes prominently red-shift. For the first mode, increasing L_2_ leads to lower absorption. In terms of absorption value and resonance frequency, there is no effect on the second resonant mode as it is only generated by L_6_. The numerical and experimental results agree well. As indicated before, Ti exhibits a small mode at around 4000 cm^−1^, which is not seen in the numerical results as an adhesion layer is not used in the simulations.

**Figure 4:**
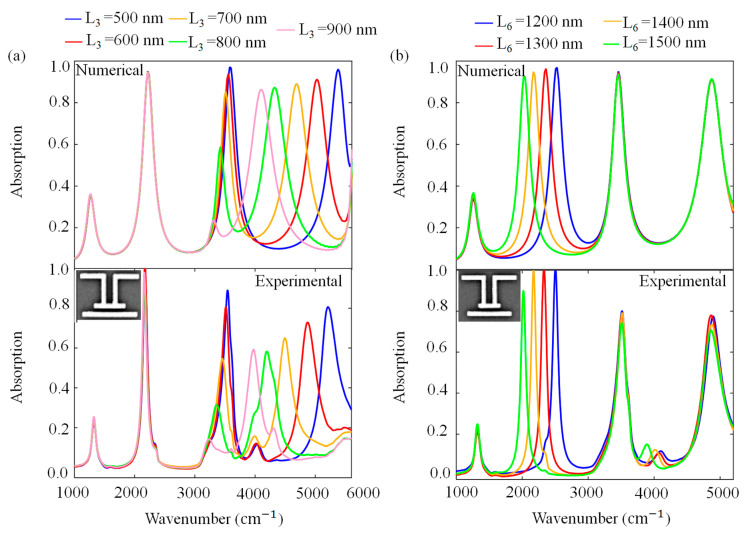
Absorption spectra of the PA platform for (**a**) numerical and experimental results of L_3_ sweep; (**b**) numerical and experimental results of L_6_ sweep under x-polarized light source. The results support the near-field enhancement distributions at various resonance frequencies. The insets show the SEM image of unit cell for L_3_ = 800 nm and L_6_ = 1200 nm. Array periodicity is 1.7 µm.

Figure 4 shows numerical and experimental parameter sweeps for L_3_ and L_6_. Figure 4a exhibits numerical and experimental absorption spectra for different L_3_ values when the other lengths are fixed at L_1_ = L_5_ = 550 nm, L_2_ = L_4_ = 700 nm, L_6_ = 1200 nm, S = 200 nm, D = 100 nm and w = 100 nm. As L_3_ increases, the fourth resonance mode significantly red-shifts. While the third mode slightly red-shifts, the absorption value of that mode reduces. There are no spectral variations in the first and second mode during the L_3_ sweep. Figure 4b presents numerical and experimental absorption spectra for different L_6_ values when the other lengths are fixed at L_1_ = L_5_ = 550 nm, L_2_ = L_4_ = 700 nm, L_3_ = 600 nm, S = 200 nm, D = 100 nm and w = 100 nm. As L_6_ is increased, only the second resonance mode significantly shifts to the lower frequencies and the second absorption value does not change in magnitude. This point also agrees well with the near-field enhancement distribution analysis of the fourth mode of the PA in Section 3.1. Discrete behavior of resonances clearly shows that electromagnetic near-field coupling between different modes is very low.

### 3.3. Refractive Index Sensitivity of the PA Platform

**Figure 5:**
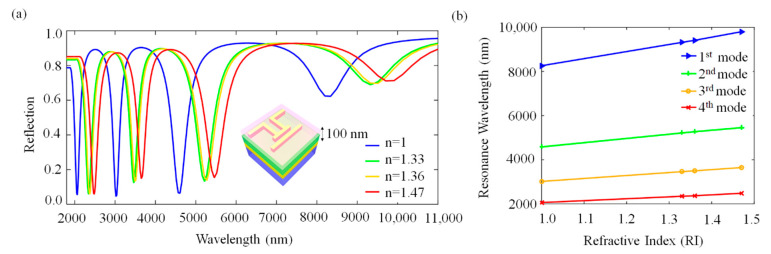
(**a**) Schematic view of embedded PA in 100 nm-thick cladding medium and reflection spectrum of PA with respect to cladding media: air (n = 1), DIW (n = 1.33), acetone (n = 1.36) and glycerol (n = 1.46). (**b**) Linear relationship between resonance wavelengths and refractive index of cladding media for the first, second, third and fourth mode show easy control and calibration of the PA for refractive index-based sensing. The corresponding device parameters: L_1_ = L_5_ = 550 nm, L_2_ = L_4_ = 700 nm, L_3_ = 600 nm, L_6_ = 1200 nm, S = 200 nm, D = 100 nm, and w = 100 nm, and array periodicity is 1.7 µm.

High near-field intensities on the PA surface indicate a potential towards high sensitivity for refractive index change over the surface environment. As the resonance frequency of the PA is related with the refractive index of the environment (air at first, n = 1), an increase in the refractive index of the surrounding media must cause a red-shift on the resonance frequency [25,26]. To investigate the refractive index sensing ability of our design, we numerically embedded the PA in different cladding media such as de-ionized water (DIW) (n = 1.33), acetone (n = 1.36) and glycerol (n = 1.46) with the thickness of 100 nm, as shown in Figure 5a. The cladding medium strongly affects the resonance characteristics of the PA by interacting with the near fields of the nanoantennas. The refractive index is formulated as n = $\sqrt{\varepsilon µ}$. At optical frequencies, magnetic permeability is nearly one. Therefore, when we chance n, the dielectric constant of the surrounding medium proportionally changes. As the refractive index increases, resonance wavelengths of the PA red-shift linearly. To show this linearity, we examined four modes and obtained the relationship between the resonance wavelength (nm) and refractive index (RI). This linear relationship can be seen in Figure 5b. As indicated in Table 1, refractive index sensitivity (S = Δλ/Δn) [25,26] values are 3251.76, 1871.39, 1335.69 and 885.46 nm/RIU for the first, second, third and fourth mode, respectively, where the RIU is the refractive index unit. This PA system supports quadruple-band resonances with full width at half maximum (FWHM) values of 1010, 400, 210 and 156 nm. The figure of merit (FOM) is the ratio of the bulk refractive index sensitivity (S) and FWHM at the corresponding resonance peak (FOM = S/FWHM) [25,26]. Therefore, we obtained FOM values of 3.23, 4.68, 6.35 and 5.67 nmRIU^−1^ for the first, second, third and fourth modes, respectively. We should note that FOM values are very close to each other for all resonances except the first mode. The reason for the first mode to have the lowest value is because gold antennas show larger FWHM for the resonances at a higher wavelength due to the optical losses in IR. Even though the surface enhancement intensities are at the same order of magnitude for each mode, one could expect to have different S due to the small near-field intensity differences and the different resonance frequencies. Therefore, such a small fluctuation of FOM is accountable. The third mode shows the highest value for FOM because it has a narrower FWHM compared to the first and the second modes. Although the fourth mode has the narrowest resonance, its refractive index sensitivity value is lower than the third mode, which associates it with a lower FOM by conventional definition. When compared to the similar studies available in the literature [37], the calculated sensitivity and FOM in this work performs well. These high sensitivities and linearity make this novel PA ideal for sensing applications from the mid- to near-IR regime of the EM spectrum.

### 3.4. Highly Sensitive Biosensing for Brain Metastasis Biomarkers

To test the biosensing capacity of the presented perfect absorber, we experimentally detected the antibody of the molecule S100A9 (anti-mouse S100A9, 73425, Cell Signaling) that was recently presented as a clinically relevant biomarker to personalize radiotherapy for patients with brain metastases [38]. The molecular mass of the S100A9 monoclonal antibody (Ab) was 14 kDa, which makes the molecule a low-molecular-mass protein that is hard to detect even when using ELISA-based immunoassays. For the immobilization of the Ab we utilized a photochemical immobilization technique (PIT). The technique relies on the control of the disulfide bridges present in the Ab by UV light that can chemically hook on the gold surface irreversibly, keeping the sites on the Ab open for antigen recognition [39,40,41]. The UV source (Trylight^®^) consisted of two U-shaped low-pressure mercury lamps (6 W at 254 nm), where a standard quartz cuvette could be easily housed. The irradiation intensity was roughly 0.3 W/cm^2^. The Ab solution in water with 15 μg/mL concentration was placed in a quartz cuvette and was exposed to UV light for 30 s. The exposed Ab was then drop casted on the sensor surface for 15 min and the sensor surface was rinsed with DI water for 15 min. The sensor was air dried. FTIR measurements with the same spectroscopic parameters indicated before were employed on the sensor surface. To check the efficiency of the Ab binding on the gold sensor surface, we first used atomic force microscopy. We measured a height difference between 2 and 3 nm on the perfect absorbers before and after the protein immobilization, which shows that the Ab on the gold surface did not accumulate, but immobilized as a monolayer. We further tested the outcome of the PIT by surface-enhanced infrared absorption spectroscopy using a different perfect absorber generated in our group and successfully spotted the amide bands [11]. Those results prove that the Ab was bonded on the perfect absorber’s gold surface successfully.

**Figure 6:**
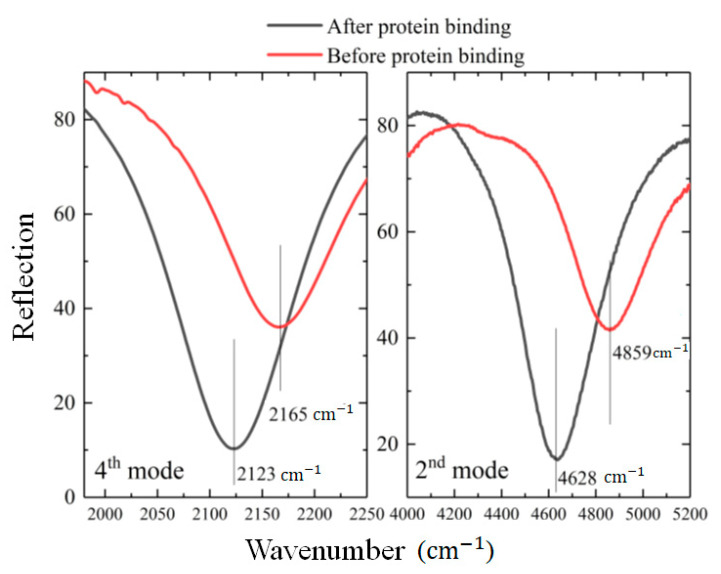
A strong red-shift after a couple of nm-thick protein binding on the PA surface is observed on fourth mode (**left**), and second mode (**right**). All the modes present a red-shift, where sensitivity is on the order of 100 nm/RIU for a 3 nm-thick protein monolayer. The results show the eligibility of the presented PA as a point-of-care device for the detection of S100A9 molecule in clinical brain metastasis studies. The corresponding device parameters: L_1_ = L_5_ = 550 nm, L_2_ = L_4_ = 700 nm, L_3_ = 600 nm, L_6_ = 1200 nm, S = 200 nm, D = 100 nm and w = 100 nm, and array periodicity is 1.7 µm.

Figure 6 shows the sensing capacity of the presented perfect absorber. All the sensors presented in Figure 3 and Figure 4 with different sizes were tested for biosensing, where the sensitivity measurements were performed at least four times for each sensor and the average results were calculated. Figure 6 shows the biosensing on the sensor with the parameters: L_1_ = L_5_ = 550 nm, L_2_ = L_4_ = 700 nm, L_3_ = 600 nm, and L_6_ = 1200 nm; the same ones that were used in Figure 5. The results show a clear red-shift for all resonance modes. Figure 6 shows the average 90 nm resonance wavelength shift for the fourth mode (~2000 cm^−1^) on the left, and the average 110 nm resonance shift for the second mode (~5000 cm^−1^) on the right. Those numbers are consistent with the fact that the fourth mode was expected to show less sensitivity compared to the second mode, as it shows lower near-field enhancements. We also observed easily detectable resonance shifts in modes one and three, in parallel to the data presented in Figure 6. The number of hotspots and enhancement factors on the antenna surface are important indicators for its sensing performance. Therefore, we preferred to use the second and the fourth modes that had the highest calculated electric near-field intensity to demonstrate the biosensing capacity of the design. The experimental sensitivity (S = Δλ/Δn) towards the Ab with RI~1.5 was calculated as ~70 for the fourth, and ~60 for the second mode. It would not be fair to compare the simulated numbers with the experimental results as the Ab used was smaller (only a few nanometers and only 14 kDa) than the cladding thickness (100 nm) used in simulations for a more general discussion. Even for such a small Ab, the sensitivity of the perfect absorber presented is high enough to detect the resonance frequency change with a naked eye. Therefore, these results clearly show the functionality of the PIT and eligibility of the presented perfect absorber as a point-of-care device for the detection of the S100A9 molecule in clinical brain metastasis studies [38].

## 4. Conclusions

In this work, we presented a quad-resonant perfect absorber operating from the mid-IR to near-IR spectra for low concentration biomolecular sensing. The optical quality of the near-unity light absorption at all resonance frequencies were discussed and explained with the near-field electric field properties. The refractive index-based sensing capacity of the PA was both theoretically and experimentally studied. The sensitivity of the resonance towards the refractive index change for the 100 nm cladding medium was calculated to be as high as 3000 nm/RIU. The photochemical immobilization technique could successfully bind the S100A9 anti-mouse antibody on the PA gold surface. The sensing of the 3 nm antibody monolayer was achieved by the designed PA with a sensitivity of 70 nm/RIU. Those results prove the capacity of the presented quad-band PA for the ultrasensitive detection of such small biomarkers in a point-of-care device to potentially personalize radiotherapy for patients with brain metastases.

## Figures and Tables

**Table 1:** Comparison of the values of refractive index sensitivity (S), full width at half maximum (FWHM) and figure of merit (FOM) of the four plasmonic modes.

Modes	S = Δλ/Δn (nm/RIU)	FWHM (nm)	FOM = S/FWHM (RIU^−1^)
First mode	3251.76	1010	3.23
Second mode	1871.39	400	4.68
Third mode	1335.69	210	6.35
Fourth mode	885.46	156	5.67

## Data Availability

Not applicable.
